# A roadmap for biocatalysis – functional and spatial orchestration of enzyme cascades

**DOI:** 10.1111/1751-7915.12386

**Published:** 2016-07-15

**Authors:** Claudia Schmidt‐Dannert, Fernando Lopez‐Gallego

**Affiliations:** ^1^ Department of Biochemistry, Molecular Biology and BiophysicsUniversity of Minnesota140 Gortner Laboratory, 1479 Gortner AvenueSt. PaulMN55108USA; ^2^ Heterogeneous Biocatalysis GroupCIC BiomaGUNEPase Miramon 182San Sebastian‐DonostiaSpain; ^3^ IkerbasqueBasque Foundation for ScienceBilbaoSpain

## Abstract

Advances in biological engineering and systems biology have provided new approaches and tools for the industrialization of biology. In the next decade, advanced biocatalytic systems will increasingly be used for the production of chemicals that cannot be made by current processes and/or where the use of enzyme catalysts is more resource efficient with a much reduced environmental impact. We expect that in the future, manufacture of chemicals and materials will utilize both biocatalytic and chemical synthesis synergistically. The realization of such advanced biomanufacturing processes currently faces a number of major challenges. Ready‐to‐deploy portfolios of biocatalysts for design to production must be created from biological diverse sources and through protein engineering. Robust and efficient multi‐step enzymatic reaction cascades must be developed that can operate simultaneously in one‐pot. For this to happen, bio‐orthogonal strategies for spatial and temporal control of biocatalyst activities must be developed. Promising approaches and technologies are emerging that will eventually lead to the design of *in vitro* biocatalytic systems that mimic the metabolic pathways and networks of cellular systems which will be discussed in this roadmap.

## Introduction

Low‐cost DNA sequencing and synthesis, high‐throughput technologies and systems level analysis of biological systems have opened the door for industrializing biology. In the coming decade, industrial biology is expected to accelerate and facilitate the production of high‐value chemicals that cannot be made by current methods or of high‐volume chemicals where biological processes are more economical and resource efficient with a much reduced environmental impact. Consequently, manufacturing of a range of chemicals and materials will increasingly utilize both enzymatic and chemical synthesis synergistically. Biomanufacturing of chemicals using cell‐based or *in vitro* methods therefore has been identified as a technology priority area in the US and Europe.

Industrial biocatalysis exploits either the metabolic capabilities of cells for the production of complex molecules from simple carbon sources or the catalytic properties of isolated enzymes for the *in vitro* synthesis of highly pure compounds from precursor molecules. While microbial production with metabolically engineered strains holds great promise for the production of commodity chemicals, synthesis of many other industrially relevant complex molecules such as fine chemicals and chemical building blocks currently is best achieved using cell‐free biocatalytic systems. A major advantage of *in vitro* systems is the ability to assemble with relative ease non‐natural biocatalytic cascade reactions by mixing and matching enzymes with the desired activities isolated from different sources (Dudley *et al*., 2015). In principal, it should be possible to reconstruct complex *in vitro* enzymatic reaction cascades that adopt the same fundamental modular design principles of cellular metabolic networks and systems. A new frontier in biocatalysis, systems biocatalysis, aims to do just this; the design of artificial metabolic networks for *in vitro* biocatalysis (Fessner 2015). Significant challenges will need to be overcome first before any of such *in vitro* systems eventually may be transformed into industrial processes. For example, biocatalyst costs (including development and production) must be reduced considerably, process efficiency increased and catalytic processes optimized for scalable downstream processing (Xue and Woodley, 2012; Ni *et al*., 2014; Wohlgemuth *et al*., 2015).

**Figure 1:**
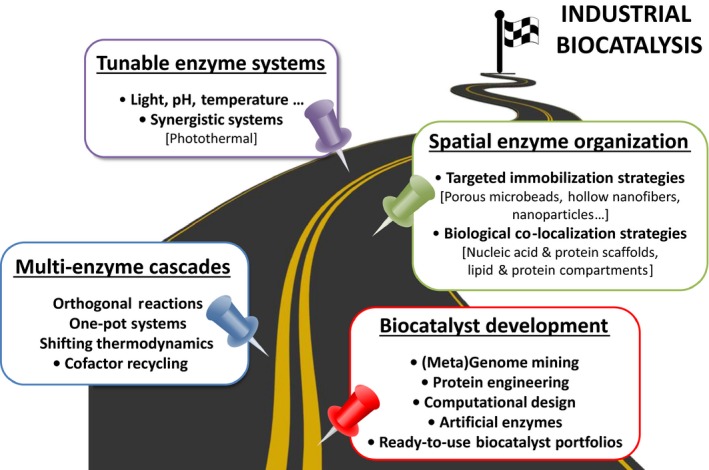
Roadmap for biocatalysis – challenges and strategies.

In this contribution, we will outline a roadmap for the next decade of industrial biocatalysis illustrated in Fig. 1. We will identify current challenges and discuss promising technologies and strategies to develop industrially relevant enzyme cascades for *in vitro* biocatalysis. We will begin with biocatalyst development and work our way to increasing complexity; from the operation of multi‐enzyme cascade reaction to their spatial organization and functional orchestration.

## Biocatalyst development

To be competitive with chemical catalysis, biocatalysis must be able to access the same or even greater chemical diversity than chemical synthesis. This can only be achieved by expanding the available repertoire of functionally diverse and robust biocatalysts that can be produced at a large scale. Currently, any biocatalytic process development begins with the identification, design and optimization of suitable biocatalysts. In the future, chemical manufacturers ideally should have access to a range of well‐characterized enzyme biocatalysts in bulk quantities that can be mixed and matched as needed for process development similar to the combination of the well‐known synthetic reactions for the design of chemical synthetic processes.

New biocatalysts can be identified from natural sources and catalytic functions optimized or changed via *in vitro* evolution or rational, structure‐based design, or a combination of both engineering strategies. While traditional screening of for example, microbial sources has led to the identification of many useful enzymes, mining of genomic sequences has unveiled many new enzymes that catalyse known reactions but with broader substrate scopes and greater selectivity. New biocatalysts may also be discovered from sources that have received little attention so far. One such source for the identification of enzymes with new and unusual activities are fungi. The increasing availability of fungal genome sequences, especially of Basidiomycota, gives access to a diverse, yet largely unexplored repertoire of oxidoreductases, oxygenases and hydrolases useful for biocatalysis (Schmidt‐Dannert, 2016). Other sources for new biocatalysts are microorganisms from extreme environments which have evolved robust enzymes that are active under harsh industrial process conditions (Ferrer *et al*., 2016).

Sourcing biological diversity for enzyme activities alone frequently is not sufficient to yield biocatalysts with the desired activities and selectivities and additional protein engineering must be applied to improve enzyme properties. Several recent examples have shown how mechanism‐guided protein engineering can transform even well‐characterized enzymes with no or low activity for a desired reaction and with poor process stability into catalysts that meet industrial requirements both in terms of productivity and operational stability (Hammer *et al*., 2015; Renata *et al*., 2015). For example, oxidoreductases have been engineered by exchanging basic active site residues with neutral residues allowing binding of amines or ketones instead of the corresponding acids. By introducing these changes into D‐aminoacid oxidase (Yasukawa *et al*., 2014) and L‐leucine dehydrogenase (Abrahamson *et al*., 2012), these enzymes have been converted into amine oxidase and alcohol dehydrogenase respectively. Both of these activities are rare in nature but highly valuable for organic synthesis.

Rational and evolutionary protein design informed by mechanistic and structural knowledge was synergistically applied for the development of a ω‐transaminase that catalyses the asymmetric amination of non‐natural ketones under industrial process conditions with high substrate and solvent concentrations. This biocatalyst was developed jointly by Merck and Codexis for the synthesis of Sitagliptin, an important drug for the diabetes treatment. Compared with the chemical process, the developed biocatalytic process increased the overall yield by 13%, productivity by 53% and reduced waste generation by 19% (Savile *et al*., 2010). Other recent examples of biocatalysts engineered with highly useful novel activities include a P450 monooxygenase catalysing diastereo‐ and enantioselective cycloproponation (Coelho *et al*., 2013) and a squalene hopene cyclase catalysing new C‐C bond formations with non‐terpenoid substrates (Hammer *et al*., 2015; Renata *et al*., 2015). Finally, structural insights into the first, bona fide Diels‐Alderase offers a path forward for the design of enzymes that perform this important C‐C bond forming reaction with a variety of substrates (Byrne *et al*., 2016).

As computational approaches become more powerful, *de novo* design of enzymes will become increasingly feasible. New active sites can be designed into suitable known protein scaffolds or the vast genomic sequence space can be screened *in silico* for suitable scaffolds based on preset active site constellations (catalaphores) for a given reaction and/or substrate binding (Steinkellner *et al*., 2014; Khersonsky and Fleishman, 2016). Alternatively, catalaphores can be designed as in the case of recent examples where organometallic complexes were tethered to an avidin protein scaffold. This “artificial enzyme” catalysed a range of non‐natural reactions such as enantioselective hydrogenation, selective oxidation of sulfides, enantioselective Suzuki reaction and metathesis reactions (Heinisch and Ward, 2015).

## Coupling enzyme activities for efficient cascade synthesis of complex molecules

To be competitive with chemical synthesis, biocatalytic processes must be robust, highly efficient and most importantly, self‐sufficient in terms of cofactor requirements and elimination of inhibitory and toxic side products. These demands can only be achieved by coupling the activities of several enzymes into cascade reactions, similar to the metabolic pathways and networks found in cells. In the last decade, dozens of enzymatic cascade reactions have been built to incorporate cofactor recycling systems that shift thermodynamic equilibriums to the final products and to eliminate *in situ* by‐products which affect both enzyme and product stability (Ni *et al*. 2014a). The architectures of these cascades is quite diverse and ranges from concurrent to sequential multi‐enzyme reactions, which in some cases are operated as orthogonal parallel cascades. These cascade reactions are designed to be self‐sufficient one‐pot reactions that operate under benign reactions conditions, with high atom economy and molecular selectivity (Sattler *et al*., 2014; Kohler and Turner, 2015; Parmeggiani *et al*., 2015). An elegant example of a simple two‐enzyme, yet industrially highly relevant reaction is the conversion of racemic mixtures of alcohols into enantiopure amines via a hydrogen‐borrowing cascade that couples the activity of an alcohol dehydrogenase with that of an engineered aminoacid dehydrogenase (Mutti *et al*., 2015). The enantiopure amines synthesized by this one‐pot bioamination cascade are important building blocks for the synthesis of many industrial chemicals.

More complex cascades are much more difficult to control and typically require significant additional optimization before they can be converted into an industrial process. For example, mono‐substituted benzenes were transformed into enantiopure L‐tyrosine derivatives in a one‐pot reaction with three concurrent enzyme reactions. Here, benzenes are first hydroxylated by an engineered P450 enzyme. In a second reaction, an engineered tyrosine phenol‐lyase catalyses a C‐C‐coupling reaction followed by an asymmetric amination of phenols with pyruvate and ammonia as co‐substrates. NADPH cofactor recycling is simultaneously performed with a glucose dehydrogenase (Dennig *et al*., 2015). Although reactions are carried out under pH control and with optimized enzyme and cofactor concentrations, less than 70% substrate conversion is achieved at preparative scale. Suboptimal substrate conversion is partially mitigated by the high regio‐ and enantioselectivity with which the cascade reaction proceeds.

Biocatalyst limitations such as pH, temperature, product, substrate or cofactor incompatibilities and/or inhibition often prevent the concurrent operation of enzymatic one‐pot cascade reactions. In chemo‐enzymatic cascades, a chemical catalyst may be poisoned by enzymatic cofactors or the catalyst and/or its reaction conditions inhibit enzyme function. The incompatible reactions then need to be performed sequentially. For example, extension of the above described L‐tyrosine cascade reaction with an oxidation/reduction cascade to synthesize the corresponding enantiopure p‐hydroxyphenyllactic acids required a sequential operation. In this extended enzyme cascade, the C‐C coupling reaction catalysed by the tyrosine phenol‐lyase is incompatible with the oxidation/reduction reactions catalysed by the second set of enzymes. (Busto *et al*., 2016). Incompatibility issues can be more dramatic for chemo‐enzymatic cascades where a chemical catalyst may be poisoned by enzymatic cofactors or the catalyst and/or its reaction conditions inhibit enzyme function. For instance, in a chemo‐enzymatic amination cascade, decoupling of the chemical and enzymatic reactions was necessary to prevent inhibition of the biocatalyst because of the high substrate load required for the chemical catalyst. In this cascade, allylic alcohol isomerization catalysed by a ruthenium complex is followed by an asymmetric amination step catalysed by an engineered ω‐transaminase to synthesize a broad range of secondary amines with excellent conversion and enantioselectivity (Rios‐Lombardia *et al*., 2015). The same ruthenium catalyst could be combined though with an alcohol dehydrogenase for concomitant one‐pot conversion of racemic mixtures of allylic alcohols to the corresponding secondary aliphatic alcohols with high enantioselectivity (Ríos‐Lombardía *et al*., 2016). In another recent example, chemical and enzyme catalyst incompatibility was solved by sequestering the chemical catalyst to prevent enzyme inhibition by the free metal complex. Here, an artificial metalloenzyme was engineered by affinity binding of a biotinylated irridum complex to a streptavidin scaffold. This metalloenzyme was then combined with a monoamine oxidase for the dynamic kinetic resolution of amines (Kohler *et al*., 2013). The protection of the organometallic catalyst within the protein scaffold prevents mutual inactivation between enzyme and chemical catalyst.

Although a range of promising bioactalytic cascade reactions have been developed, demonstrating the feasibility of carrying out complex one‐pot reaction schemes at preparative scale (g l^−1^ isolated products), many challenges still remain. The above examples illustrate that both enzyme choice and reaction engineering are critical for achieving robust cascade reactions that operate concurrently and at maximal performance. Therefore, in addition to developing a functionally diverse portfolio of robust, ready‐to‐use biocatalysts, engineering of optimal reaction environments for the operation of multi‐enzymatic cascades – much like the operation of metabolic pathways in cellular systems – will be critically important for realizing advanced biocatalytic processes suitable for the chemical industry.

## Spatial organization of robust enzyme cascades

Cells operate a multitude of multi‐enzyme cascade reactions simultaneously with high efficiency, while at the same time controlling metabolic fluxes, preventing the build up of toxic intermediates and directing metabolites to the correct enzymatic pathways. Key to the optimal function of cellular metabolic networks is the spatial organization and temporal control of multi‐enzymatic cascade reactions of metabolic pathways. Spatial organization within cells is achieved through a range of different mechanisms (reviewed in: Conrado *et al*., 2008; Proschel *et al*., 2015). On a micro‐scale, enzymes are compartmentalized into membrane‐bound or protein‐based organelles, while on a nano‐scale, enzymes form supramolecular complexes and/or are tethered to membranes, the cytoskeleton or protein scaffolds. By bringing catalysts in close proximity, the efficiency of enzymatic cascade reactions can be significantly increased and the thermodynamic equilibrium shifted to the final product (Huang *et al*., 2001). In such assemblies, a high local concentration of a substrate is immediately converted into a product that then becomes the substrate of the next enzyme and so on. This sequential channelling of substrates from enzyme to enzyme also prevents the accumulation of intermediates that may be toxic, reactive, form dead‐end shunt products or are picked up as substrates of undesirable, competing enzymatic reactions. The same design principles for spatial organization of metabolic enzymes may be adapted to create robust and highly efficient cell‐free biocatalytic cascade reactions that operate concurrently in one‐pot. With proper design, it should become possible to build complex biocatalytic networks were for example, several cascades operate in parallel to provide building blocks for downstream cascades, much like the different precursor pathways that feed into biosynthetic pathways.

Enzyme immobilization on solid support is a commonly used strategy to increase the efficiency of multi‐enzymatic cascades (Garcia‐Galan *et al*., 2011; Jia *et al*., 2014). Immobilization strategies, however, typically have been designed for only one or two reaction steps because co‐immobilizing several enzymes on the same support is not trivial (Maurer *et al*., 2003; Rocha‐Martin *et al*., 2012). Current co‐immobilization strategies require careful selection of the immobilization chemistry and of the type of carrier in order to preserve the activity and stability of all enzymes involved in the biocatalytic system. Increasingly, new materials are used for more biocompatible, site‐specific immobilization of recombinant biocatalysts and to mimic the spatial design strategies of biological systems. Solid support matrices now provide affinity groups for interactions with peptide tags (e.g. His‐tag, streptavidin‐tag or charged peptide tags) added to the biocatalysts. For example, a seven‐enzyme pathway for *de novo* production of UDP‐galactose was successfully immobilized onto nickel agarose beads (Liu *et al*., 2002). Likewise, three His‐tagged biosynthetic enzymes were successfully immobilized onto CdSe‐ZnS quantum dots to generate a multi‐enzyme complex for menaquinone biosynthesis *in vitro* (Kang *et al*., 2014). In another example, surface‐activated hollow nanofibres filled with polyelectrolytes were used to anchor a five‐enzyme system and retain cofactors for the conversion of CO_2_ to methanol with high yields and concomitant cofactor recycling (Ji *et al*., 2015).

In the past 5 years, biological enzyme co‐localization mechanisms are increasingly investigated and engineered for metabolic pathway engineering and *in vitro* applications. Enzymes can be co‐localized (i) using natural or synthetic protein scaffolds and nucleic acid scaffolds (Lee *et al*., 2012; Horn and Sticht, 2015; Siu *et al*., 2015), (ii) by compartmentalizing into protein‐based microcompartments or capsids (Worsdorfer *et al*., 2011; Held *et al*., 2013, 2016; Kim and Tullman‐Ercek, 2013; King *et al*., 2014; Lai *et al*., 2014; Bobik *et al*., 2015; Azuma *et al*., 2016; Kerfeld and Melnicki, 2016) and organelles (Avalos *et al*., 2013; Zecchin *et al*., 2015; DeLoache *et al*., 2016; Giessen and Silver, 2016), (iii) by attaching multi‐enzyme systems to membranes (Raghupathy *et al*., 2015) or (iv) the generation of supramolecular assemblies (Tan *et al*., 2015).

The majority of the co‐localization and compartmentalization systems developed thus far have been developed for *in vivo* applications with the goal of increasing the efficiency of metabolic pathways. Each of the two strategies, co‐localization and compartmentalization, has its challenges: scaffolds ideally should allow control over the co‐localization and proximity of the enzymes in cascade, while encapsulation of enzymes into compartments should likewise be programmable. Co‐localization onto protein or DNA‐scaffolds currently relies almost exclusively on reversible interactions, which could lead to disassembly *in vivo* and diminished robustness for *in vitro* applications. Furthermore, the low number of binding domains currently available limits the number of enzymes that can be co‐localized in a controlled fashion. Successful examples of scaffold‐based co‐localization include the design of a synthetic scaffold using protein domain interactions from eukaryotic signalling protein for programmable co‐localization of three mevalonate pathway enzymes to increase terpenoid biosynthesis (Dueber *et al*., 2009). In other examples, nucleic acid scaffolds were designed for the programmable co‐localization of enzymes or proteins *in vivo* using either DNA‐ (Conrado *et al*., 2012) or RNA‐binding domains (Sachdeva *et al*., 2014; Adamala *et al*., 2016).

Notable *in vitro* systems that mimic substrate channelling of metabolic pathways include a two‐enzyme cascade tethered to a DNA‐scaffold (Fu *et al*., 2014) and a three‐enzyme artificial metabolosome that was built by using the cohesin‐, dockerin‐ and cellulose‐binding domains (the latter for immobilization on cellulose) from bacterial cellulosomes (You and Zhang, 2013). The DNA‐tethered enzymes catalysed the asymmetric reduction of oxaloacetate into L‐malic acid with *in situ* recycling of the NADH cofactor also tethered to the DNA‐scaffold as a swinging arm between the two enzymes. Cascade‐efficiency could be increased 300 times by optimizing the DNA‐scaffold architecture to control proximity and ratios of tethered catalysts and cofactor (Fu *et al*., 2014). Likewise, proximity of the three enzymes docked to the designed artificial cellulosome significantly increased reaction rates and yields of fructose 1,6‐biphosphate from glucose‐6‐phosphate compared with the soluble enzyme system (You and Zhang, 2013). Stable supramolecular enzyme assemblies can also be achieved by genetically fusing catalysts to self‐assembling proteins. In an elegant example, a P450 and its two electron transfer proteins were assembled into a water insoluble, reusable biocatalytic gel using a heterotrimeric protein as a scaffold (Tan *et al*., 2015).

As an alternative to scaffolds, bacterial protein microcompartments and smaller capsids are intensely studied for their use in biotechnology. So far only a virus capsid has been engineered to encapsulate enzymatic cascade reactions (Patterson *et al*., 2014; Glasgow *et al*., 2015). Bacterial microcompartments are larger than virus capsids and have evolved to compartmentalize metabolic pathways (e.g. the ethanolamine or propanediol degradation pathways in *Salmonella*, or RuBisco in cyanobacteria) in bacteria. Encapsulation peptides target metabolic enzymes into the compartments during their self‐assembly (Lawrence *et al*., 2014; Aussignargues *et al*., 2015; Held *et al*., 2016). Pores formed by the self‐assembling shell proteins connect the metabolically active lumen of the compartments with the exterior, which is the cytoplasm in bacterial cells. These compartments can be heterologously produced and non‐native cargo proteins targeted to their interior (Held *et al*., 2013, 2016; Bobik *et al*., 2015; Kerfeld and Melnicki, 2016). In principle, these compartments may be engineered as robust *in vitro* nano‐bioreactors with functionalized shell‐surfaces for immobilization, orthogonal encapsulation mechanisms and pore selectivities tailored for specific cascade reactions.

Another area of intense research is the encapsulation of enzymes into synthetic vesicles and more recently polymersomes have been used to entrap complex multi‐enzymatic systems such as the transcription/translation machineries of cells and other metabolic cascades The ultimate goal of these efforts is to built artificial cells that can function as chemical microreactors that are equipped with the ability to produce their own enzymes, facilitate transport across their membrane and potentially communicate with other vesicles performing the same or a different task (Vriezema *et al*., 2007; van Dongen *et al*., 2009; Caschera and Noireaux, 2014; Elani *et al*., 2014; Miller and Gulbis, 2015; Kuchler *et al*., 2016).

The examples so far have shown that spatial organization of enzymes and cofactors significantly impacts the efficiency of enzymatic cascade reactions. A number of innovative and promising platforms are currently explored. For industrial applications, robustness, cheap production costs and adaptability need to be at the centre for the design of such systems. It remains to be seen if nucleic acid scaffolds can withstand industrial process conditions. The design of modular and programmable enzyme cascades will require the development of more bio‐orthogonal covalent targeting mechanisms, such as the Spy/SnoopTag and Spy/SnoopCatcher systems (Reddington and Howarth, 2015; Veggiani *et al*., 2016).

## Tunable *in vitro* enzyme cascades

Achieving temporal control of *in vitro* multi‐enzyme cascade reactions represents yet another major challenge in the design of complex biocatalytic systems that mimic cellular system. The introduction of artificial actuators into co‐localized multi‐enzyme cascades will afford precise temporal control of catalytic units and modules depending on process requirements. Actuators should modulate the activity and selectivity of enzymes in response to environmental changes such as pH, temperature or light. Conjugation of synthetic macromolecules to proteins already has been successfully used to modulate the function of proteins and enzymes in response to environmental cues (Cobo *et al*., 2015). Controlling protein function using light is the simplest and most efficient method and such control mechanisms have been applied both for *in vivo* and *in vitro* applications (Knor, 2009). For example, conjugation of a azobenzene group to a cysteine residue located in the substrate‐binding pocket of a lipase allowed light‐dependent control of both activity and selectivity of the enzyme (Bautista‐Barrufet *et al*., 2014). Bioconjugation of proteins with thermo‐responsive polymers was used to switch glucose oxidase activity on and off in response to temperature changes. Here, temperature induced changes in polymer packing caused blockage of the enzyme active site (Shimoboji *et al*., 2003). Because of the required heating and cooling of the reaction system, temperature‐based control, however, is much slower compared with light‐induced control. Photothermal materials therefore have been combined with enzymes where illumination exerts a thermal effect that controls enzyme activity under specific illumination conditions. For example, glucokinase was co‐entrapped with gold nanorods in alginate particles; near infrared light illumination of the nanomaterial then caused an increase in temperature surrounding the enzyme which in turn accelerated enzyme activity (Blankschien *et al*., 2013).

These examples show that researchers are beginning to develop new and innovative systems to control enzyme activities. Efforts in optogenetics are expected to provide new tools that may be adapted to tune and control the activities of engineered catalysts both *in vitro* and *in vivo* (Schmidt and Cho, 2015).

## Conclusion

The realization of complex biocatalytic cascade reactions that can be performed cost‐efficiently at a scale required for industrial applications faces many challenges that can only be addressed by increasing cross‐disciplinary research efforts in biological engineering, biochemistry, chemistry and process engineering. Advances in synthetic biology, materials science as well as protein engineering have created exciting new tools and approaches that must be merged to create robust systems and catalysts with which to orchestrate (chemo)enzymatic cascades that execute synthetic tasks akin to the metabolic networks of living cells.
